# Interrupted inferior vena cava with deep vein thrombosis

**DOI:** 10.1259/bjrcr.20220100

**Published:** 2022-12-08

**Authors:** Matan Kraus, Noam Tau

**Affiliations:** 1 Department of Diagnostic Imaging, Sheba Medical Center, Ramat Gan, Israel; 2 Sackler School of Medicine, Tel Aviv University, Tel Aviv, Israel

## Abstract

A 22-year-old healthy man presented to the emergency department with worsening left flank and testicular pain. Lower abdominal pain and lower urinary tract symptoms, were also noted.

Contrast-enhanced CT demonstrated several vascular malformations: both common iliac veins converging to an infrarenal inferior vena cava (IVC) with an absent cephalad IVC. Multiple collateral veins were noted, and both the Azygos and Hemiazygos veins were seen dilated, serving as an alternative venous drainage path due to the interrupted IVC.

The patient’s CT was also notable for several pathologies: bilateral iliac vein thrombosis and left-sided testicular vein thrombus with surrounding fat stranding, suggestive of testicular vein thrombophlebitis.

The patient was admitted, and received antibiotic and anticoagulation treatment, with clinical improvement.

Hypercoagulability work-up was obtained, and the patient was found to be heterozygous for Factor V Leiden.

Interrupted IVC with azygos continuation is an uncommon, and mostly a benign vascular malformation, resulting from abnormal development of IVC-contributing segments during embryogenesis. It is associated with lower limb deep vein thombosis and hypercoagulable states. It is imperative for radiologists to be acquainted with this entity, in order to avoid misdiagnosis.

Testicular vein thrombosis is uncommon, mostly associated with prothrombotic disorders, and it should be considered when coagulopathy is suspected.

## Clinical presentation

A 22-year-old, previously healthy male presented to our emergency department (ED) complaining of left flank, groin and testicular pain. Lower abdominal pain and lower urinary tract symptoms, including increased urine frequency and urgency were also noted. The patient was then discharged with antibiotic treatment for suspected urinary tract infection (UTI). Four days later, while still receiving treatment, the patient had a repeat visit to the ED with worsening symptoms.

During his initial ED visit, vital signs were normal and the patient did not have fever. Physical examination was unremarkable, apart from painful percussion of the left flank.

Blood tests showed mild elevation of C-reactive protein (CRP) to 19.4 mg/l (normal range = 0–5 mg/l) and normal white blood count (WBC) of 7.6 K/microL (normal range = 4–10.8 K/microL). Spot urinalysis was positive for mild proteinuria (25 mg/Dl, normal range = 0–1 mg/Dl), and negative for hematuria and leukocyturia.

On ultrasound (not shown here), mild left-sided hydronephrosis was noted, without hydroureter.

The patient was discharged with a prescription for oral antibiotic therapy of 100 mg Nitrofurantoin * 3 /day for 5 days, for suspected UTI.

Four days later, as the patient returned to the ED, fever of 38ºC and tachycardia up to 120 beats per minute were noted. Physical examination was again positive for left flank pain, and this time erythema of the left thigh was noticed as well. On palpation, the left testicle was mildly tender and had an absent cremasteric reflex, but was not swollen.

Blood tests revealed an elevated CRP level (97.7 mg/l), while WBC count remained within the normal range. Spot urinalysis was now positive for mild hematuria (50 erythrocytes/UL, normal range = 1–2 erythrocytes/UL), but negative for proteinuria or leukocyturia. Mid-stream urine culture obtained during the prior ED visit was positive for mixed colonies, suggestive of contaminants rather than true bacterial infection.

## Imaging findings

Contrast-enhanced CT of the abdomen and pelvis was performed for suspected renal stones, and to assess for any other source of the patient’s presentation.

**Figure 1.**
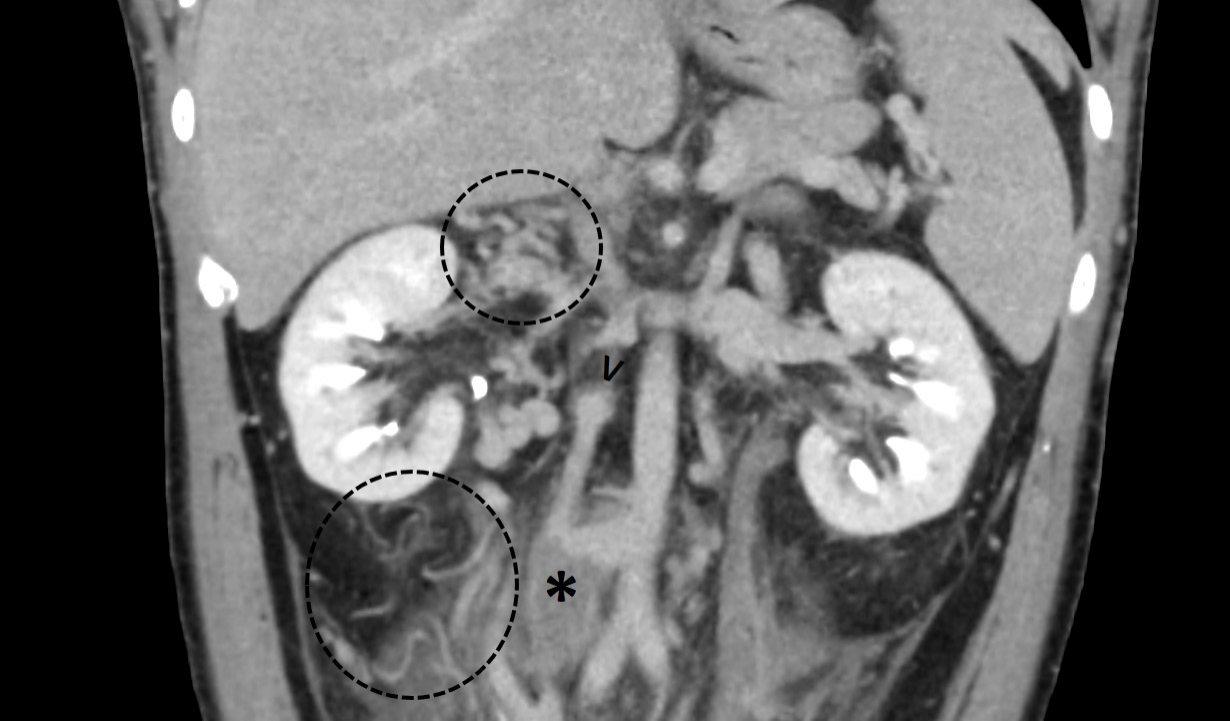
MIP coronal reconstruction of a contrast-enhanced CT, demonstrating the IVC interruption (arrowhead), thrombus in the IVC (asterisk), and multiple collateral veins in the retroperitoneum (dashed circles). IVC, inferior vena cava; MIP, maximum intensity projection.

**Figure 2.**
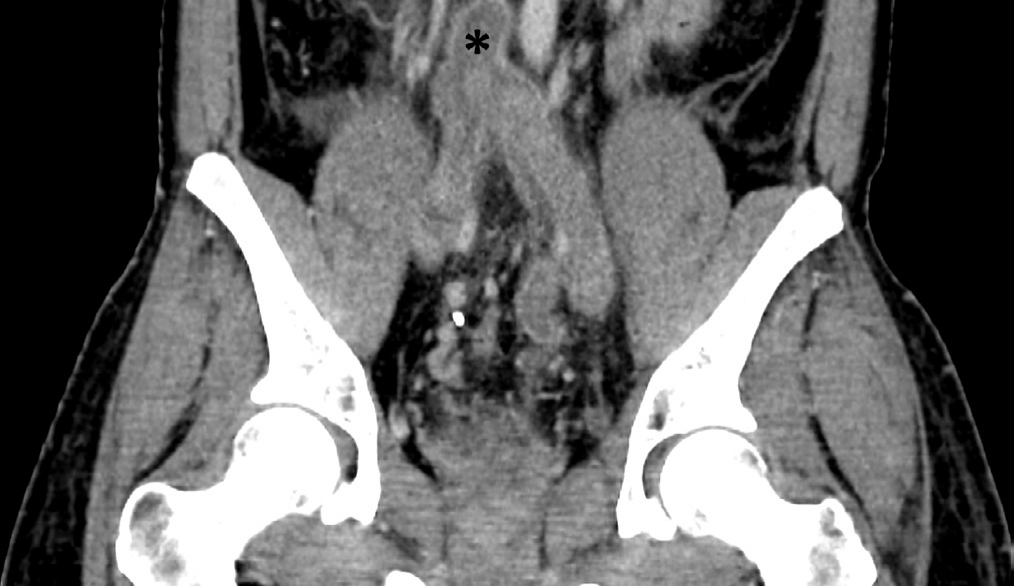
Contrast-enhanced CT, coronal reformat, showing the thrombus in the Inferior Vena Cava cephalad to the iliac veins converging (asterisk).

**Figure 3.**
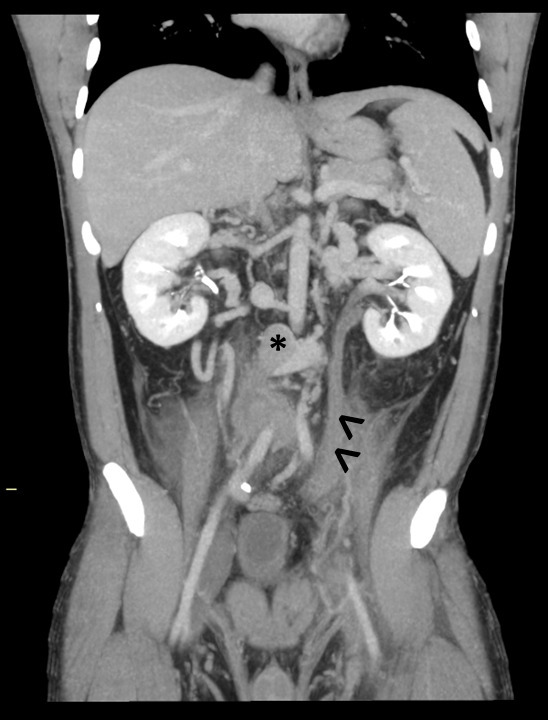
MIP coronal reconstruction of a contrast-enhanced CT, demonstrating the occluded left testicular vein (arrowheads), with adjacent fat stranding, compatible with thrombophlebitis. MIP, maximum intensity projection.

**Figure 4.**
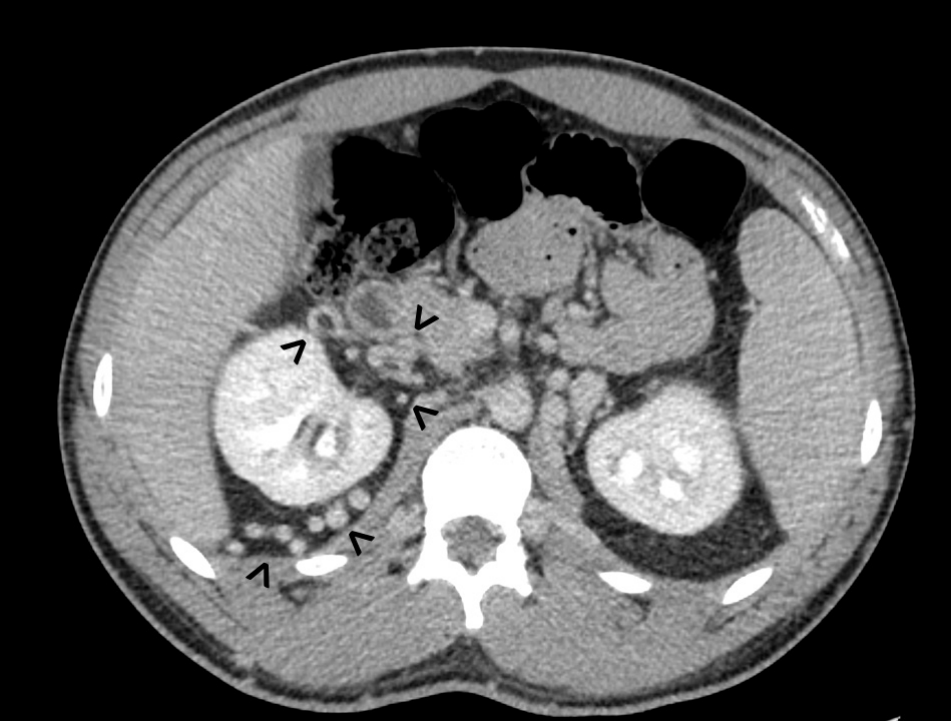
Multiple collateral veins in the retroperitoneum (arrowheads), serving as the caudal alternative venous drainage route due to the interrupted IVC. IVC, inferior vena cava.

**Figure 5.**
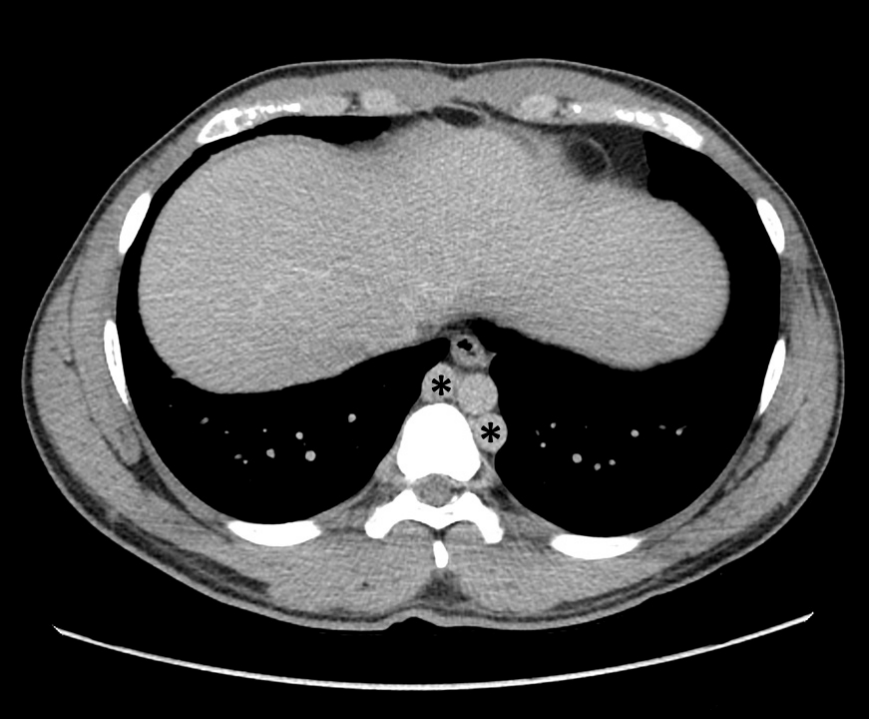
CT at the level of the lower chest, demonstrating dilation of both Azygos and Hemiazygos veins (asterisks), serving as the cephalad alternative venous drainage route due to the interrupted IVC. IVC, inferior vena cava.

The CT was negative for nephrolithiasis, but demonstrated several other anomalies and pathologies: Both common iliac veins were seen converging to an infrarenal inferior vena cava (IVC) with an absent cephalad IVC (Figures 1 and 2 and Figure 3, asterisk); multiple collateral veins, mostly in the retroperitoneum (Figure 4, arrowheads); dilated azygos and hemiazygos veins were seen accompanying the thoracic aorta (Figure 5, asterisks), serving as an alternative venous drainage path due to the interrupted IVC. To accompany all those findings, a filling defect was seen in the left testicular vein with surrounding fat stranding (Figure 3, arrowheads), compatible with thrombophlebitis.

Lower limb and scrotal ultrasound with Doppler demonstrated left ilio-femoral-popliteal deep vein thrombosis (DVT) and right popliteal DVT (not shown here). Testicular ultrasound was normal.

The clinical and imaging data led to the diagnosis of left testicular vein thrombophlebitis and bilateral lower extremity DVT, in a patient with a congenital interrupted IVC accompanied by azygos and hemiazygos continuation.

## Treatment, outcome, follow-up

Following a multidisciplinary discussion between abdominal and invasive radiologists, vascular surgeons, hematologists and emergency medicine physicians, it was decided to treat the patient non-invasively.

The patient received intravenous Ampicillin and Gentamicin treatment alongside low molecular-weight heparin, with rapid clinical improvement.

Repeat urinalysis and urine culture were now normal with no bacterial growth, and therefore the urinary symptoms were thought to be secondary to urinary tract irritation by the testicular venous thrombophlebitis rather than frank UTI.

Following discharge, the patient began follow-up in the outpatient hematology clinic. Hypercoagulability work-up was obtained on his first visit, and the patient was found to be heterozygous for Factor V Leiden. Subsequently, low molecular-weight heparin treatment was continued.

## Discussion

During embryogenesis, the IVC is the product of formation, regression, and fusion of three pairs of veins: the supracardinal, subcardinal and posterior cardinal. This complex development makes the IVC prone to anatomic malformations.^1,2^

Interrupted IVC with azygos continuation is an uncommon malformation.^3^ The prevalence of IVC anomalies ranges between 0.07 and 8.7% in the general population, while the prevalence of azygos or hemiazygos continuation is about 1.5%.^4^ These anomalies are usually asymptomatic, and usually diagnosed incidentally when imaging is performed for other reasons.^4^

Acquaintance with these uncommon malformations and their diagnosis is important for two main reasons: first, familiarity with these anatomic variants can help to avoid misdiagnosis when encountered.^3^ Second, in the operative and invasive settings, it is crucial to be aware of these malformations, as it can affect pre-procedural planning, such as choosing an entry point for catheterization.^3^ In cases where an anomaly is accompanied by predominant venous drainage by the azygos route, intraprocedural preservation of the azygos is of paramount importance, since accidental ligation of this draining vein in these patients may lead to catastrophic results.^4^

IVC interruption is regarded as a predisposing factor to the development of lower extremity DVT.^5^ Moreover, it was observed that lower limb DVT is more common in males, and for the most part is proximal and bilateral.^6^

Absence of IVC was estimated to be present in 5% of unprovoked DVT cases in young patients (age <30 years).^7^ Furthermore, it was recommended to consider an IVC anomaly in young patients presenting with otherwise idiopathic lower limb DVT, such as in our patient.^6^

A study by Sagban et al have showed a higher prevalence of thrombophilias (*e.g.* Factor V Leiden heterozygosity, mutation in MTHFR gene and homocysteinemia) in patients with IVC interruption. Nevertheless, the authors considered the IVC malformation as the main risk factor for DVT, and addressed the coagulopathies as a mere contributing factor to DVT in those patients.^6^

Testicular vein thrombosis is an uncommon entity, first reported by Gavin in 1935.^8^ The little data that exist in the literature stems from numerous case reports.^9,10^ As in our patient, most reported cases occurred in the left gonadal vein.^11^

Several etiologies for gonadal vein thrombosis in these patients have been described, including coagulopathies, such as protein C deficiency or factor V Leiden mutation, underlying cancer, iatrogenic catheterization complications, inguinal trauma and secondary to nutcracker syndrome.^11^

In a case series published in 2017, testicular vein thrombosis was shown to be strongly associated with intra-abdominal and retroperitoneal malignancies.^10^ Therefore, it was suggested to inquire for an underlying malignancy, though no formal guidelines have been issued.^9^

Testicular vein thrombosis poses a true clinical diagnostic challenge, since the absence of specific clinical findings can easily lead to other pathologies.^9^ Doppler ultrasound is considered the gold-standard diagnostic test, but in some cases, CT is imperative, in order to assess the complete thrombus extension.^9,11,12^ Of note, those imaging studies are only ordered if clinical suspicion is high enough to warrant those, and therefore, although rare, this entity should be part of the differential diagnosis in unilateral testicular pain.

There is no consensus as to the treatment of choice in testicular vein thrombosis. Still, in most cases, the current limited literature is inclined to apply non-invasive treatment, including anticoagulant and anti-inflammatory medications. Surgical treatment is preferred when the etiology is of more urgent nature, *e.g.* in ruling out complicated incarcerated inguinal hernia.^9,11,12^

In conclusion, IVC interruption is an uncommon, and for the most cases, a benign vascular anomaly. It is imperative for clinicians and radiologists to be familiar with this entity, in order to avoid misdiagnosis, safely adjust invasive treatment and be aware of the association with hypercoagulable states. Testicular vein thrombosis is a rare entity, and a difficult diagnosis to make due to its unspecific presentation. When assessing acute scrotum, it is important to take this etiology into account, especially if a coagulopathy is suspected.

## Learning points

- IVC interruption is rare, and usually asymptomatic.
- Nonetheless, it may first present as lower limb DVT, and it is associated with a high prevalence of thrombophilias.
- Familiarity with this anatomic variant can assist in both avoiding misdiagnosis and prompt early diagnosis and treatment.
- Testicular vein thrombosis is a rare etiology for acute scrotum, and poses a diagnostic challenge, due to its unspecific and vague clinical presentation.
- Still, it should be taken into account, particularly when coagulopathy is suspected.
